# Cost–Effectiveness Analysis of Pharmacist Adherence Interventions in People Living with HIV/AIDS in Pakistan

**DOI:** 10.3390/healthcare11172453

**Published:** 2023-09-01

**Authors:** Ali Ahmed, Juman Abdulelah Dujaili, Lay Hong Chuah, Furqan Khurshid Hashmi, Long Khanh Dao Le, Zeenat Fatima Chatha, Saval Khanal, Ahmed Awaisu, Nathorn Chaiyakunapruk

**Affiliations:** 1 Monash University Health Economics Group (MUHEG), School of Public Health and Preventive Medicine, Monash University, 553 St Kilda Road, Melbourne, VIC 3004, Australia; 2 School of Pharmacy, Monash University, Jalan Lagoon Selatan, Bandar Sunway, Subang Jaya 47500, Selangor, Malaysia; 3 Riphah Institute of Pharmaceutical Sciences, Riphah International University, Islamabad 44000, Pakistan; 4 Swansea University Medical School, Singleton Campus, Swansea University, Wales SA1 8EN, UK; 5 Punjab University College of Pharmacy, University of Punjab, Allama Iqbal Campus, Lahore 54000, Pakistan; 6 Department of Community Medicine and Global Health, University of Oslo, 0318 Oslo, Norway; 7 Health Economics Consulting, Norwich Medical School, University of East Anglia, Norwich NR4 7TJ, UK; 8 Department of Clinical Pharmacy & Practice, College of Pharmacy, QU Health, Qatar University, Doha 2713, Qatar; 9 College of Pharmacy, University of Utah, Salt Lake City, UT 84112, USA; 10 IDEAS Center, Veterans Affairs Salt Lake City Healthcare System, Salt Lake City, UT 84108, USA

**Keywords:** pharmacist, adherence, health outcomes, cost–effectiveness, incremental cost–effectiveness ratio, Pakistan

## Abstract

**Background:** Evidence has shown the positive impact of pharmacist involvement on the adherence and health outcomes of people living with HIV/AIDS. However, whether such intervention provides value for money remains unclear. This study aims to fill this gap by assessing the cost–effectiveness of pharmacist interventions in HIV care in Pakistan. **Methods:** A Markov decision analytic model was constructed, considering clinical inputs, utility data, and cost data obtained from a randomized controlled trial and an HIV cohort of Pakistani origin. The analysis was conducted from a healthcare perspective, and the incremental cost–effectiveness ratio (ICER) was calculated and presented for the year 2023. Additionally, a series of sensitivity analyses were performed to assess the robustness of the results. **Results:** Pharmacist intervention resulted in higher quality-adjusted life years (4.05 vs. 2.93) and likewise higher annual intervention costs than usual care (1979 USD vs. 429 USD) (532,894 PKR vs. 115,518 PKR). This yielded the ICER of 1383 USD/quality-adjusted life years (QALY) (372,406 PKR/QALY), which is well below the willingness-to-pay threshold of 1658 USD (446,456 PKR/QALY) recommended by the World Health Organization Choosing Interventions that are Cost-Effective. Probabilistic sensitivity analysis reported that more than 68% of iterations were below the lower limit of threshold. Sensitivity analysis reported intervention cost is the most important parameter influencing the ICER the most. **Conclusion:** The study suggests that involving pharmacists in HIV care could be a cost-effective approach. These findings could help shape healthcare policies and plans, possibly making pharmacist interventions a regular part of care for people with HIV in Pakistan.

## 1. Background

Approximately 39 million people were living with the human immunodeficiency virus (HIV) or acquired immunodeficiency syndrome (AIDS) in 2022 [1]. More than 50% of people living with HIV/AIDS (PLWHA) are from low- and middle-income countries (LMICs) [1,2]. Pakistan, located in Asia and the fifth most populated country, has around 240,000 PLWHA [3]. The number of new HIV infections has been growing steadily, reaching 25,000 in 2020, while it was 14,000 in 2010 [4]. The deaths related to AIDS have had a tremendous increase from 1400 in 2010 to 8000 in 2020 [3]. As of June 2022, only 58% of the registered PLWHA were following their ARVs treatment properly [5]. The way the epidemic is spreading has changed, moving from mostly affecting certain groups to affecting the whole population through sexual networks [4]. Out of all PLWHA, 53% belong to key groups, including men who have sex with men (25%), people who use drugs (24%), female sex workers (2%), and transgender individuals (2%) [3].

Safe and effective antiretroviral therapy (ART) is available for the treatment of HIV/AIDS, and with proper ART adherence, PLWHA can live longer and healthier, like a normal person [6,7]. Adherence to antiretrovirals (ARVs) suppresses viral load (VL) to undetectable levels, increases CD4 T lymphocyte count, and improves the quality of life [6,8]. Despite the availability of free ARVs, approximately 27% of PLWHA worldwide do not adhere to ARVs [1]. Non-adherence to ARVs is a major problem, and it is even worse in LMICs such as Pakistan, where it is 44% [3,6]. Adherence to ART constitutes a complex behavior influenced by both socioeconomic and cultural factors [9]. Socioeconomic elements encompass income, education, and healthcare access, thereby impacting the consistency of treatment, particularly for those with limited resources [10]. Cultural beliefs play a pivotal role in shaping perceptions of health and the acceptability of treatment, exhibiting variations across societies [11]. Forgetfulness and the demanding nature of adhering to timed medication intake contribute to non-adherence, as does the presence of fatigue and associated side effects that affect an individual’s motivation [12]. Feelings of hopelessness stemming from the chronic nature of the illness, coupled with the stigma surrounding HIV, can act as deterrents to adherence [13]. Concerns related to non-disclosure of HIV status hinder openness and the effective management of medication, while religious beliefs can wield influence over medical decision-making [14,15]. Recognizing and comprehending these multifaceted influences are of paramount importance in the development of targeted interventions aimed at fostering successful adherence [6,9,16].

Pharmacists are ideally placed in hospitals and community pharmacy settings, and they are accessible healthcare providers who have demonstrated that they can effectively contribute to resolving HIV/AIDS therapy adherence issues [17,18,19]. Pharmacists are increasingly involved in the management of medication therapy for PLWHA, with the aim of optimizing the use of medications and improving health outcomes [20,21,22]. Pharmacist interventions can play a crucial role in mitigating medication errors, improving medication literacy skills, and promoting adherence to medication therapy [21,23,24,25]. In addition, medication therapy management services can help patients better manage their chronic conditions, leading to fewer complications and a reduced need for acute care services. This can ultimately result in lower healthcare costs and improved patient outcomes [22,26]. A recent meta-analysis found pharmacist care interventions in HIV care had a positive impact on medication adherence (odds ratio OR: 2.70 [95%, confidence interval CI 1.80, 4.05]), VL suppression (OR: 4.13 [95% CI 2.27, 7.50]), and CD4 count improvement (median difference: 66.83 cells/mm3 [95% CI 44.08, 89.57]) when compared to usual care [17].

Cost–Effectiveness analyses (CEA) have become an important tool to help decision makers better understand how to deliver healthcare efficiently [27,28,29,30]. CEA provide valuable information on the clinical and economic trade-offs resulting from the allocation of healthcare resources for a population [31,32]. Recently, a randomized controlled trial (RCT) evaluated the effectiveness of pharmacist intervention in addition to usual care in HIV care in Pakistan and found intervention significantly increased the adherence and CD4 count in the intervention arm, but no study has explored the cost–effectiveness of pharmacist intervention in HIV care [33]. In order to implement pharmacists in HIV/AIDS care, it is imperative to conduct the CEA from a healthcare perspective, as all HIV care is provided free of cost to all PLWHA in Pakistan [2,4,9]. Pakistan is an LMIC with limited resources, and there was a gap in the data on whether pharmacist intervention could provide a good value for the scarce healthcare dollars that would need to be allocated to its provision. As HIV/AIDS care continues to evolve, it is important to demonstrate the value of clinical professional services provided by pharmacists. This will aid in facilitating public funding decision-making and ensure the long-term sustainability of such services for PLWHA. Therefore, the present study evaluated whether involving pharmacist adherence to educational and counseling sessions in addition to usual care will efficiently use scarce resources.

## 2. Methods

Methodological guidance for this study was taken from the recommendations from the Second Panel on Cost–Effectiveness in Health and Medicine [34], and the Consolidated Health Economic Evaluation Reporting Standards (CHEERS 2022) checklist [35] was used for proper reporting (Supplementary Materials).

### 2.1. Intervention Trial

We used data from a single-blinded randomized controlled trial (RCT) undertaken between September 2018 and February 2019 at the HIV care center, Pakistan Institute of Medical Sciences (PIMS), Islamabad, Pakistan [33]. Eligible patients were HIV-positive, greater than 18 years of age, and had been taking antiretroviral therapy (ART) for more than 3 months. A total of 128 patients living with HIV/AIDS were initially screened for inclusion in the randomized controlled trial (RCT), out of which 100 individuals were shortlisted as they met the inclusion criteria [33]. From this group, 34 patients were lost to follow-up due to various reasons such as refusal to participate, relocation, loss to follow-up, and death. As a result, the study ultimately included a total of 66 patients living with HIV/AIDS, with 33 in each group; intervention and control. The intervention group consisted of 23 males and 10 females, while the usual care group included 18 males, 10 females, and 5 transgender individuals. In brief, 33 PLWHA received only usual care, and 33 received usual care plus a single session of 30 min pharmacist education and counseling session. The pharmacist education was focused on disease knowledge, ARVs effectiveness, side effects, ARVs resistance, CD-4 count, VL, diet, supplementary herbs, and safe sex information. The counseling component focused on barriers to ARV use, as reported by PLWHA. Its aim was to assist participants in comprehending their medication-taking behaviors while acknowledging the actions required to maintain a high level of adherence. After two months (8 weeks) follow-up, pharmacist intervention increased adherence and CD-4 count [33].

### 2.2. Model Structure

**Figure 1:**
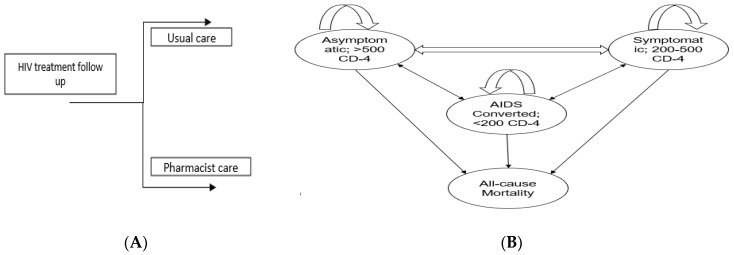
(**A**) Shows the overall approach to model structure. People living with HIV/AIDS (PLWHA) will either go through usual care or pharmacist care depending on randomization (**B**) Show a Markov model with four stages: stage A, also known as the Asymptomatic stage; stage B, the Symptomatic stage; stage C, representing the AIDS-converted stage; and stage D, indicating the Death stage. A Markov model was employed to determine the transition probabilities between stages of HIV/AIDS. Upon entering the care programs, PLWHA were allocated to specific stages based on their CD4 count. Every two months, PLWHA were re-evaluated and transitioned between stages if there were changes in their CD4 count.

We performed cost–effectiveness analysis of pharmacist interventions based on decision analytical modelling as it is difficult to conduct lifetime longitudinal trial due to recurrent disease complications and budget constraints [36]. Furthermore, a Markov modeling is useful to extrapolate the benefit of pharmacist interventions over one year instead of 2-month follow-up in the trial. The RCT length was only two months, so to keep the modeling results realistic and avoid over-extrapolation, we kept the time horizon length of one year. HIV primarily targets T-helper cells or CD4 T-lymphocyte cells, a type of white blood cell that plays an important role in the immune system. Once the virus enters the body, it attaches to the CD4 receptor and enters into T-lymphocyte cells, hijacks their machinery to make copies of itself, and destroys the cells in the process, producing thousands of copies of the virus [37]. The continuous attack on CD4 cells by the new viruses will eventually lead to the depletion of the CD4 lymphocyte cells, a crucial part of the immune system. As the CD4 cells decrease, the immune system becomes weaker, making it harder for the body to fight infections and illnesses. This weakening of the immune system is what leads to the development of AIDS (Acquired Immunodeficiency Syndrome). The model was structured as a 4-state Markov model, with each health state defined by the World Health Organization (WHO) staging system for HIV (Figure 1). The four health states were as follows: stage A (asymptomatic with a CD4 count of >500 cells/mm^3^), stage B (symptomatic with a CD4 count of 200–500 cells/mm^3^), stage C (AIDS-defining illness with a CD4 count of <200 cells/mm^3^), and stage D (death). These health states capture the key changes in CD4 cell count associated with costs and quality of life. PLWHA could change between health states every 2-month cycle. We set the 2-month cycle length based on the Chatha et al. trial duration [33]. We estimated the one-year clinical and economic outcomes of pharmacist intervention. This was calculated in terms of the incremental cost–effectiveness ratio (ICER) and quality-adjusted life years (QALYs). According to the findings of Chatha and their team [33], the intervention led to increased CD-4 counts for more than 50% of the participants by the end of the trial. This improvement positively impacted the health status of individuals with PLWHA as they advanced from stage C to stage B. Moreover, a portion of the participants also moved from stage B to the more favorable stage A.

### 2.3. Model Inputs and Data Sources

Model stage probabilities and transitional probabilities were obtained from the Pakistani-origin RCT and HIV Pakistan cohort [3,33]. Death probability was not available from Pakistan, so it was taken from England and Wales’ national HIV/AIDS data [38]. Utility values were calculated from a Pakistani study that assessed the PLWHA health-related quality of life (HRQoL) by using Euroqol 5 dimensions (EQ-5D-3L) [6]. As per Ahmed et al., asymptomatic (stage A) utility is 0.83, symptomatic (stage B) utility is 0.34, and AIDS converted (stage C) utility is 0.03 [6]. Each stage has its own cost of treatment. In stages B and C, the cost is generally high for the ARVs and depends on the comorbid conditions’ treatments. For this study, we determined the cost of each stage in consultation with HIV/AIDS physicians and NACP of Pakistan data (Table 1). Costs are provided within a range, acknowledging that in certain instances, they could be lower or higher than the specified range.

The United States dollar (USD) conversion to Pakistani rupee (PKR) was taken as 1 USD = 270 PKR [39]. The cost of pharmacist intervention was calculated based on expert opinion and researchers’ familiarity with implementing pharmacists in a Pakistani clinical setting. Salary information was sought by consulting with pharmacists working in both government (10 individuals) and private hospitals (10 individuals). In the RCT, PLWHA was attended by one time in two months. First, pharmacist session was around 30 min, and the follow-up sessions were about 15 min. The average salary for a pharmacist in Pakistan is approximately 80,000 PKR/month (290 USD/month). (Ranging between 40,000–120,000 PKR/month [137–414 USD/month]). With monthly salary, pharmacist is bound to work 48 h/week and can provide services up to 448 PLWHA (range 400–800 PLWHA)/month. Results were summarized as the average direct cost per participant per 8-week duration of the trial. The cost for each HIV disease stage was determined in consultation with NACP experts, and this cost estimation was applied to both the intervention and usual care groups. Further details are given in the table.

We conducted the analysis from the standpoint of healthcare and did not discount effectiveness and cost because the length of the analysis was only one year, 2023.

### 2.4. Willingness to Pay

To evaluate the cost–effectiveness of pharmacist interventions in HIV care, a willingness-to-pay (WTP) threshold was used to determine whether the results are cost-effective. In some countries, such as the United Kingdom (UK) and Thailand, fixed WTP thresholds are employed to make coverage decisions [40]. WTP threshold has not been developed in Pakistan. The WHO does not have a fixed stance on WTP thresholds. However, the Commission on Macroeconomics and Health has suggested that dynamic thresholds for LMICs, 3x national gross domestic product (GDP) per capita per additional QALY gained, may be reasonable [40,41]. In 2022, Pakistan’s GDP per capita was 1658 USD [42], conservatively chosen as WTP threshold in this study, and 3x the GDP of Pakistan is 4974 USD.

### 2.5. Statistical Analysis

The statistical analysis was performed by using the TreeAge Pro 2021 software (TreeAge Pro 2021, R1.1; TreeAge Software, LLC, Williamstown, MA, USA). The cost–effectiveness was calculated in the form of ICER, as defined by the incremental costs (the difference in costs between the intervention group and the usual care group) relative to QALY. Net monetary benefit (NMB) in CEA represents the difference between the monetary value of health benefits gained from an intervention and its associated costs, helping to determine whether the intervention is financially advantageous compared to alternatives or a reference point. To avoid ambiguous interpretation of the ICER, the NMB as defined by incremental QALYs multiplied by the threshold minus the incremental costs was calculated as

NMB = (incremental benefit × threshold) − incremental cost

If the NMB was equal to or greater than zero, intervention would be considered cost-effective.

### 2.6. Sensitivity Analysis

The purpose of sensitivity analysis in cost–effectiveness analysis is to gauge the influence of varying input parameters on the outcomes, thereby examining the stability and reliability of the results under different scenarios [43]. A series of one-way sensitivity analyses were conducted by varying (within limits) the input parameters and observing the resulting changes in the output to find the parameters that significantly influence the results and which are less important [44,45]. We varied the utility and cost of pharmacist intervention by ±20%. The results of the one-way sensitivity analysis are displayed in the tornado diagram.

### 2.7. Probabilistic Sensitivity Analysis (PSA)

PSA was conducted to assess the uncertainty in base case analysis by allowing all variables to change simultaneously by assigning a probability distribution [46,47]. Beta distribution was assigned to utilities, and gamma distribution was assigned to cost data. The Monte Carlo simulation method with 1000 iterations was performed to construct a scatter plot and cost–effectiveness acceptability curve. The results are presented in a scatter plot, where the incremental effect of each analysis is plotted on the *x*-axis and the incremental cost on the *y*-axis. The curve was plotted to determine the probability that the pharmacist intervention group was cost-effective across a range of WTP thresholds.

## 3. Results

### 3.1. Base Case Analysis

Using the trial data, we found pharmacist intervention yielded higher QALYs than usual care (4.05 vs. 2.93) and likewise higher annual intervention costs than usual care (1979 USD vs. 429 USD) (532,894 PKR vs. 115,518 PKR), resulting the ICER of a 1383 USD/QALY (372,406 PKR/QALY). Given that the Pakistan cost–effectiveness threshold is 1658 USD/QALY (446,456 PKR/QALY), having pharmacist participation in PLWHA treatment had an ICER less than the Pakistan threshold compared with usual care. NMB of pharmacist intervention was 4748 USD (1,278,514 PKR) compared to 4440 USD (1,195,578 PKR) of usual care.

### 3.2. Sensitivity Analysis

**Figure 2:**
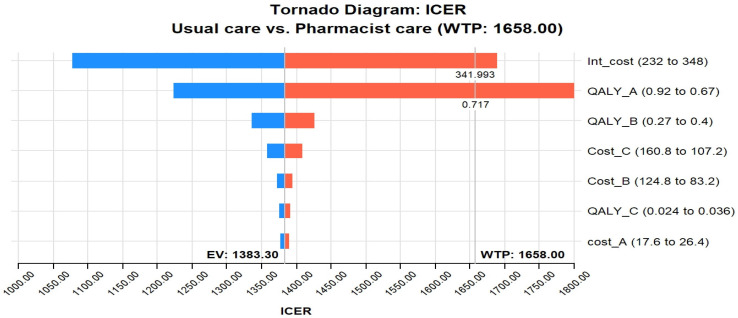
Tornado diagram of pharmacist care in HIV care compared with usual care. Abbreviations used: ICER, incremental cost–effectiveness ratio; WTP, Willing to pay; QALY_A, quality-adjusted life year-stage A utility; Int, Intervention; EV, Expected value.

The tornado diagram in Figure 2 shows that the most influential parameter in the CEA of pharmacist interventions in HIV care is the intervention cost and QALY utility of stage A. The value of these two variables was potent enough to be above the GDP per capita of Pakistan, indicating that the cost–effectiveness of pharmacist interventions in HIV care is highly dependent on the cost of the intervention and the utility scores of stage A. For example, if the intervention cost crosses 341.9 USD, it will cross the threshold; likewise, if the utility of stage A crosses 0.71, it will cross the WTP. In addition, the tornado diagram shows that other influential parameters include the cost of stage B and stage C infections. This implies that the cost–effectiveness of pharmacist interventions in HIV care is also influenced by the costs associated with managing HIV-related complications and the disease’s advanced stages (B and C).

### 3.3. Probabilistic Sensitivity Analysis

**Figure 3:**
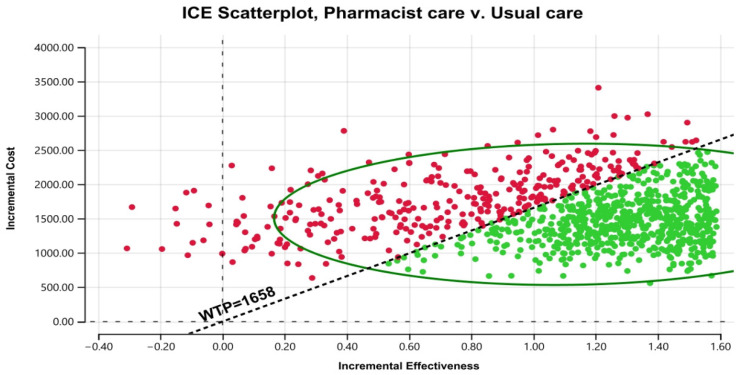
Scatter plot of pharmacist care in HIV/AIDS treatment compared with usual care on a cost-effective plane. ICE stands for incremental cost–effectiveness. WTP stands for willingness to pay.

**Figure 4:**
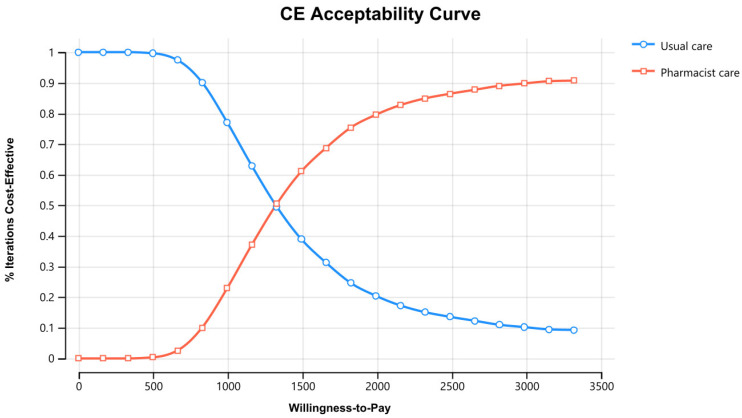
Cost–Effectiveness acceptability curve of pharmacist care in HIV/AIDS care compared with usual care. CE stands for cost–effectiveness.

Figure 3 displays the cost–effectiveness plane with a graphical representation of 1000 iterations. The majority of the estimated ICERs (68%) were in the plane’s north-east quadrant, below the cost–effectiveness threshold. Considering parameter uncertainty, this means that the intervention had a higher associated cost and greater health benefits than usual care. Figure 4 depicts the cost–effectiveness acceptability curve. The pharmacist intervention was cost-effective 52% of the time, 68% of the time, and 90% of the time at WTP thresholds of 1383 USD/QALY, 1658 USD/QALY, and 2898 USD/QALY, respectively. Values found indicated that all WTP values were within the 3x GDP of Pakistan.

## 4. Discussion

This study represents the first attempt at evaluating the cost–effectiveness of pharmacist care, involving education and counseling, in individuals living with HIV/AIDS (PLWHA). The primary findings shed light on the potential benefits of incorporating pharmacists into HIV care. The study’s core outcomes reveal that the inclusion of pharmacists in the care of PLWHA leads to both higher costs and higher Quality-Adjusted Life Years (QALYs) in comparison to the usual care approach. Specifically, the study’s analysis yielded an Incremental Cost–Effectiveness Ratio (ICER) of 1383 USD/QALY (equivalent to 372,406 PKR/QALY) for the pharmacist intervention, a value below the accepted cost–effectiveness threshold of 1658 USD/QALY (446,456 PKR/QALY) considered as a conservative willingness-to-pay (WTP) threshold in Pakistan. This suggests that the pharmacist intervention could be considered cost-effective given this threshold, indicating that the additional costs associated with the intervention are justifiable considering the gained QALYs. The sensitivity analysis conducted further highlighted the significance of two key variables: the cost of the intervention itself and the utility of stage A. The study indicates that if the cost of the intervention surpasses 341.9 USD, the intervention’s cost–effectiveness would exceed the baseline threshold. Likewise, if the utility of stage A crosses a threshold of 0.71, it would result in crossing the WTP threshold. In essence, this study underscores the potential value of pharmacist involvement in HIV care by demonstrating the positive trade-off between higher costs and improved health outcomes. The findings suggest that pharmacist care can be deemed cost-effective within the context of the chosen WTP threshold, providing valuable insights for healthcare decision-makers and stakeholders considering the integration of pharmacist interventions into HIV care strategies.

A two-month RCT conducted by Chatha et al. reported that adherence and CD-4 count significantly improved in the pharmacist intervention arm [33]. Our study corroborates this finding and indicates that with subsequent pharmacist interventions, more people living with HIV/AIDS (PLWHA) will transition from stage C to stage B and from stage B to stage A in the pharmacist intervention group compared to the usual care group. These outcomes led to a reduction in the costs linked to stages C and B. It is important to note that these costs can differ from person to person based on factors such as age and comorbid conditions. These results are consistent with the growing body of evidence supporting the integration of pharmacists into healthcare teams [48,49,50,51,52,53]. The study by Shrestha et al. in 2021 showed that involving pharmacists in a multidisciplinary team can have significant benefits in terms of HIV prevention and cost savings. Specifically, the study found that the pharmacist-led intervention averted 2.75 HIV transmissions and saved 1.28 million USD and 12.2 quality-adjusted life years (QALYs) [54]. Moreover, Dilworth et al. cost analysis found that pharmacist intervention total cost of 22,779 USD, less than the future savings in averting HIV-related medical care expenditure of 67,351 USD (2022) [21]. Transitioning from the AIDS stage (stage D) to the asymptomatic stage (stage A) in HIV care offers several advantages, including improved quality of life with fewer symptoms and complications, reduced medical costs by minimizing the need for intensive interventions, and a lowered risk of mortality. Additionally, individuals in the asymptomatic stage often have lower viral loads, contributing to reduced disease transmission, while the improved response to treatment enhances both physical and psychological well-being.

As most of the data inputs are from Pakistan, findings are ready to be implemented in Pakistan and can be generalized to countries with similar demographics. Each year, many pharmacists are produced in Pakistan, but the problem is their clinical practice training [55,56]. Before pharmacist implementation, pharmacists must receive extensive training in disease epidemiology, treatment, prevention, pharmacotherapy, strategies to overcome adherence barriers, treatment outcomes, health education, effective communication skills, patient counseling techniques, and HRQoL assessment skills [17]. NACP of Pakistan can consider the development of a collaborative clinical practice manual and engage pharmacists after providing training to the pharmacists in infectious diseases or HIV/AIDS care in their HIV treatment guidelines. Using CEA evidence, the NACP of Pakistan can prioritize and allocate resources to interventions that have been shown to be cost-effective in preventing or treating HIV/AIDS, while avoiding interventions that are not cost-effective. This can help ensure that the available resources are used in the most efficient way possible, maximizing the benefits of the program.

Our CEA has several strengths; we obtained transitional probabilities from Pakistani trial data and the HIV cohort established by the NACP of Pakistan. Second, cost data were taken from the Pakistan perspective in consultation with pharmacists working in the govt and private sector and the NACP of Pakistan. Third, utility values were also entirely taken from the Pakistani study. These data inputs demonstrate that our findings are unique and should be useful for policymakers considering involving pharmacists in HIV/AIDS care in Pakistan, as inputs clearly reflect the Pakistani context.

Our CEA also has some limitations, such as the death probabilities were not available in the trial and Pakistan HIV cohort, so we used the national HIV-infected cohort for England and Wales [38]. Therefore, the data might be different across the regions due to demographic variations. Second, we obtained the cost of intervention data from a general survey through calls and emails to the pharmacists working in government and private settings working in different disease specialties. As of now, pharmacists are not part of HIV care, so in the future, a large investigation involving quantitative and qualitative surveys should be designed to better determine the cost of a pharmacist visit. Third, HIV stage costs were derived from ARV cost and PIMS hospital Islamabad data. Most of the hospitalization costs and major surgeries for comorbid conditions are covered through the Sehat sahulat program of Pakistan [57]. As a consequence, those excluded costs would not be significantly different between the pharmacist and usual care group. In the sensitivity analysis, we addressed this limitation by trying to change 20% of the costs of stages. Fourth, as pharmacists are also proven to provide antiretroviral stewardship [23], future research should focus on longer-term RCTs with an antiretroviral stewardship component alongside counseling and education as well as cost–effectiveness analysis to further check the intervention impact on HIV prevention, and cost saving.

## 5. Conclusions

The outcomes of our research strongly indicate that interventions involving counseling and educational support by pharmacists yield significantly enhanced health advantages for individuals facing the challenges of living with HIV/AIDS. However, it is worth noting that these interventions do entail a higher cost compared to the standard care approach. Furthermore, our study underscores the fact that the involvement of pharmacists in such interventions remains cost-effective even when assessed against widely accepted willingness-to-pay (WTP) benchmarks. This economic viability suggests that integrating pharmacist-led interventions into HIV/AIDS care strategies holds the potential to yield substantial benefits. The implications of our study extend beyond the realm of research. We believe that these findings should serve as a catalyst for meaningful discussions among pharmacists and public policy stakeholders. The discourse should revolve around the redefinition of the scope of practice and reimbursement policies, thereby facilitating a more extensive engagement of pharmacists in the landscape of HIV/AIDS care. Such collaborative efforts have the capacity to foster improvements in the overall quality of care offered to individuals living with HIV/AIDS. By promoting pharmacist involvement and encouraging policy adjustments, we can collectively advance the standard of care, enhance patient outcomes, and contribute to a more holistic approach to managing this critical healthcare challenge.

## Figures and Tables

**Table 1:** Inputs plugged in the model.

Parameters	Distribution	Base Case	Plausible Range	Sources
Transition Probabilities	Beta	Trial data	Trial data	Chatha et al. [33] Simmons et al. [38] NACP et al. [3]
**Utilities**				
Asymptomatic (>500 CD-4)	Beta	0.83	0.67–0.99	Ahmed et al. [6]
Symptomatic (200–500 CD-4)	Beta	0.34	0.27–0.40	Ahmed et al. [6]
AIDS Converted (<200 CD-4)	Beta	0.03	0.024–0.036	Ahmed et al. [6]
**Cost (2023 USD)**				
Intervention cost	Gamma	290	232–348	Assumptions
Stage A, Asymptomatic	Gamma	22	17.6–26.4	NACP et al. [3]
Stage B, Symptomatic	Gamma	104	83.2–124.8	NACP et al. [3]
Stage C, AIDS converted	Gamma	134	107.2–160.8	NACP et al. [3]

USD: United States dollar; NACP: National AIDS Control Program of Pakistan.

## Data Availability

All relevant data sets used for this study are appropriately cited and reported within the study.

## Supplementary Materials

The following supporting information can be downloaded at: https://www.mdpi.com/article/10.3390/healthcare11172453/s1, CHEERS 2022 Checklist.
