# Supplementary material for: Cost–Effectiveness Analysis of Pharmacist Adherence Interventions in People Living with HIV/AIDS in Pakistan
Source: Healthcare (Basel). 2023 Sep 1;11(17):2453. doi: 10.3390/healthcare11172453 (PMC10487586; doi:10.3390/healthcare11172453)
Supplement: Supplementary file 1 [file healthcare-11-02453-s001.zip › healthcare-2545216-supplementary.pdf]

CHEERS 2022 Checklist

Topic	No.	Item	Location where item is reported
**Title**			
	1	Identify the study as an economic evaluation and specify the interventions being compared.	Title
**Abstract**			
	2	Provide a structured summary that highlights context, key methods, results, and alternative analyses.	Abstract
**Introduction**			
**Background and objectives**	3	Give the context for the study, the study question, and its practical relevance for decision making in policy or practice.	Background all three paragraphs
**Methods**			
**Health economic analysis plan**	4	Indicate whether a health economic analysis plan was developed and where available.	Methods: Subheading; Model structure
**Study population**	5	Describe characteristics of the study population (such as age range, demographics, socioeconomic, or clinical characteristics).	Methods: Subheading; Intervention Trial
**Setting and location**	6	Provide relevant contextual information that may influence findings.	Methods: Subheading; Intervention Trial, Model structure
**Comparators**	7	Describe the interventions or strategies being compared and why chosen.	Methods: Subheading; Model structure
**Perspective**	8	State the perspective(s) adopted by the study and why chosen.	Sub heading; Model Inputs and data sources last two lines
**Time horizon**	9	State the time horizon for the study and why appropriate.	Sub heading; Model Structure
**Discount rate**	10	Report the discount rate(s) and reason chosen.	Sub heading; Model Inputs and data sources last two lines

Topic	No.	Item	Location where item is reported
**Selection of outcomes**	11	Describe what outcomes were used as the measure(s) of benefit(s) and harm(s).	Methods subheading; Interventional Trial
**Measurement of outcomes**	12	Describe how outcomes used to capture benefit(s) and harm(s) were measured.	Methods subheading; Model structure
**Valuation of outcomes**	13	Describe the population and methods used to measure and value outcomes.	Methods subheading; Model structure
**Measurement and valuation of resources and costs**	14	Describe how costs were valued.	Methods sub heading Model Inputs and data sources
**Currency, price date, and conversion**	15	Report the dates of the estimated resource quantities and unit costs, plus the currency and year of conversion.	Methods sub heading Model Inputs and data sources
**Rationale and description of model**	16	If modelling is used, describe in detail and why used. Report if the model is publicly available and where it can be accessed.	Model structure
**Analytics and assumptions**	17	Describe any methods for analysing or statistically transforming data, any extrapolation methods, and approaches for validating any model used.	Methods: Statistical analysis
**Characterising heterogeneity**	18	Describe any methods used for estimating how the results of the study vary for subgroups.	sensitivity analysis
**Characterising distributional effects**	19	Describe how impacts are distributed across different individuals or adjustments made to reflect priority populations.	Methods, Last paragraph
**Characterising uncertainty**	20	Describe methods to characterise any sources of uncertainty in the analysis.	Methods, Last paragraph
**Approach to engagement with patients and others affected by the study**	21	Describe any approaches to engage patients or service recipients, the general public, communities, or stakeholders (such as clinicians or payers) in the design of the study.	Not applicable
**Results**			
**Study parameters**	22	Report all analytic inputs (such as values, ranges, references) including uncertainty or distributional assumptions.	Results, first paragraph figures and Tables

Topic	No.	Item	Location where item is reported
**Summary of main results**	23	Report the mean values for the main categories of costs and outcomes of interest and summarise them in the most appropriate overall measure.	Results, second paragraph
**Effect of uncertainty**	24	Describe how uncertainty about analytic judgments, inputs, or projections affect findings. Report the effect of choice of discount rate and time horizon, if applicable.	Results, third paragraph
**Effect of engagement with patients and others affected by the study**	25	Report on any difference patient/service recipient, general public, community, or stakeholder involvement made to the approach or findings of the study	Not reported
**Discussion**			
**Study findings, limitations, generalisability, and current knowledge**	26	Report key findings, limitations, ethical or equity considerations not captured, and how these could affect patients, policy, or practice.	Discussion
**Other relevant information**			
**Source of funding**	27	Describe how the study was funded and any role of the funder in the identification, design, conduct, and reporting of the analysis	End of manuscript
**Conflicts of interest**	28	Report authors conflicts of interest according to journal or International Committee of Medical Journal Editors requirements.	End of manuscript

From: Husereau D, Drummond M, Augustovski F, et al. Consolidated Health Economic Evaluation Reporting Standards 2022 (CHEERS 2022) Explanation and Elaboration: A Report of the ISPOR CHEERS II Good Practices Task Force. Value Health 2022;25. doi:10.1016/j.jval.2021.10.008
